# Social learning in swarm robotics

**DOI:** 10.1098/rstb.2020.0309

**Published:** 2022-01-31

**Authors:** Nicolas Bredeche, Nicolas Fontbonne

**Affiliations:** Sorbonne Université, CNRS, Institut des Systèmes Intelligents et de Robotique, ISIR, F-75005 Paris, France

**Keywords:** evolutionary robotics, collective robotics, swarm robotics, on-line distributed reinforcement learning, social learning

## Abstract

In this paper, we present an implementation of social learning for swarm robotics. We consider social learning as a distributed online reinforcement learning method applied to a collective of robots where sensing, acting and coordination are performed on a local basis. While some issues are specific to artificial systems, such as the general objective of learning efficient (and ideally, optimal) behavioural strategies to fulfill a task defined by a supervisor, some other issues are shared with social learning in natural systems. We discuss some of these issues, paving the way towards cumulative cultural evolution in robot swarms, which could enable complex social organization necessary to achieve challenging robotic tasks.

This article is part of a discussion meeting issue ‘The emergence of collective knowledge and cumulative culture in animals, humans and machines’.

## 1. Introduction

Swarm robotics is about collectives of robots that coordinate to collectively accomplish a task, even though interactions between robots are local [1,2]. A robot swarm is composed of robots with limited communication and computation capabilities, which allows us to limit the construction cost, but also requires us to design individual behaviours that result in a self-organized collective. It is important to note that swarm robotics refers to a particular *class* of collective robotics, but not to a particular kind of collective *behaviour* (e.g. actual swarming is only one possible collective behaviour among many others).

Swarm robotics is considered as a promising candidate for a large range of applications ranging from agriculture, emergency and rescue, warehouse management, surveillance, exploration and environmental monitoring (see [3] for a review of targeted applications). Recent advances in software and hardware may shortly lead to a surge in the deployment in industrial applications, as envisioned by Dorigo and colleagues [4], even if research in this field is relatively young.

As early as the 1990s, Maja Mataric showed that it was possible to accomplish a certain number of classical tasks with several robots without oversight control, such as homing, foraging, and moving in groups [5]. Each robot interacts with its immediate neighbours, either physically or by exchanging messages, and the actions taken individually combine to create an organization at a macroscopic scale, which is neither observable nor measurable by a single robot. The rules that produce a particular behaviour are constructed by hand by trial-and-error, and are sometimes inspired by behaviours observed in social insects or groups of animals.

Work conducted at Harvard under the direction of Radhika Nagpal uses these same principles, and has shown that it is possible to program robots to self-assemble into a certain predefined shape [6] or to collectively build a 3D structure, transporting bricks to relevant locations [7]. Very recently, swarm robotics has become increasingly popular, as illustrated by the recent publication of several works on this topic in major journals [8–11]. In particular, the work of [12] shows that the material limitations in terms of communication distance between robots can be advantageous when the environment is dynamic and requires a fast detection of environmental changes.

However, it is not always easy to identify which self-organized collective behaviours will be effective in solving a problem, nor to define and program the individual behaviours that will allow a global organization to emerge at the swarm level. Indeed, a swarm of robots is a complex system by definition, involving individuals whose interactions are difficult to model (e.g. because of the complexity of physical interactions between robots [8,13]). Machine learning—in particular, reinforcement learning methods inspired by natural evolution [14–16]—can automate the design of individual behavioural strategies, provided that it is possible to measure the performance of the swarm on the desired task (e.g. the efficiency measured in a foraging task in terms of the quantity of resources retrieved). This is a classical approach in robotics, where learning is performed in a centralized way by an omniscient computer, which allows us to propose a solution as a behavioural strategy followed by each robot. The learned strategy is then deployed in an operational situation without any further learning, as global monitoring and evaluation are no longer available. Despite the various problems of using controlled environments during learning [10], this form of prior-to-deployment learning has been successfully applied to several problems, from training builder robots for construction [7] to autonomous high sea boats for surveillance [17] and outdoor UAVs reconstructing a communication network after a natural disaster [18].

The approach we present in this paper focuses on a particular case of learning for swarm robotics, where learning starts when robots are deployed in real life, and not before, thus removing the necessities for a controlled laboratory environment. This is a class of problems where the conditions for performing a task are not known *a priori*, and where learning is supported only through local communication between robots [19].

The learning algorithm is then built on interactions between robots: each robot performs actions that depend on its immediate experience of the world and can copy all or part of the decision strategies put in place by the robots it encounters. The best-rated strategies are thus diffused in the swarm, in a process that resembles social learning as observed in nature [20], but in a robotic context.^1^

While social learning in nature relies on imitation between individuals according to observable characteristics (age, health, majority, etc.), the goal here is to use learning to maximize performance on a task initially defined by the human designer (e.g. foraging, patrolling, group movement, etc.). Moreover, the goal is to discover behavioural strategies that maximize the swarm performance, and not just the individual performance. This is actually a major challenge of social learning in swarm robotics, as swarm performance is not directly accessible by individual robots.

In this paper, we aim at providing an introduction to social learning in swarm robotics as a particular type of machine learning method, both presenting the general concepts and the details of a practical implementation, as well as presenting future challenges and inspirations for the field. The paper outline is as follows: we first describe the basic principles of social learning for swarm robotics (§2), which we illustrate with a foraging task where the algorithmic implementation of social learning in a robot swarm is thoroughly described and analysed (§3). Then, we discuss the current challenges of social learning in swarm robotics, and how these artificial learning methods relate to social learning in nature (§4).

## 2. Elements of social learning in swarm robotics

The class of problems that can be addressed by social learning in swarm robotics is framed by three constraints on locality:

— local interaction: robots are situated in the environment. As such, each robot has a local experience of the environment, both in terms of accessible information and possible actions. As robots are physical entities, this also implies that complex dynamics can emerge from local interactions, such as robots colliding with each other when the density of robots is too high, which can be detrimental (e.g. robots are stuck) or beneficial (e.g. robots physically align with one another, which can be used for collective motion [8]).
— local communication: robot-to-robot communication is limited in radius, whether because of technical limitations (short-range communication apparatus) or by design (constrained communication can be beneficial in dynamic environments [12]). Moreover, there may be limitations in terms of the quantity of information that can be transferred. This means that diffusion over the swarm takes time, as message hopping from one robot to another is not instantaneous. Even if all robots are packed together, computational limitations make it unlikely that all robots can share all information at the same time. On the positive side, local communication means that social learning can be conducted by exchanging information (such as parameter values of a decision module) rather than solely relying on imitation through observation.
— local performance self-assessment: there is no central coordinator that estimates and communicates a particular robot’s contribution to the group. This means that each robot carries its own self-assessment mechanism, which is used to estimate the robot’s contribution to the task from the swarm perspective. This self-assessment function is provided by the human supervisor before deployment, and is used by each robot thereafter. It is designed to reward individual strategies that are relevant to global performance, but may be partly inaccurate due to the limited perception of each robot. As an example, maximizing the foraging performance at the individual level is (generally) aligned with maximizing foraging at the level of the swarm. However, too many foraging robots in too small an area can lead to an overcrowding effect, implying that some robots are better off standing aside so that the swarm as a whole is more efficient.

The third aspect of locality is where the *artificial* diverges from the natural. While in nature the ‘performance’ of a particular individual may be evaluated in terms of her/his ability to survive and reproduce, this is not the case here. In swarm robotics, performance assessment is explicitly calculated by an *ad hoc* evaluation function, designed prior to deployment by a human supervisor with a task in mind. Hence, a robot may be measured as efficient with respect to its participation to the accomplishment of the task, without its own integrity being taken into account. This has a major implication for the dynamics of social learning, as the very definition of successful behavioural strategies now depends both on their ability to diffuse over the population, and their ability to perform well on a user-defined task.

**Figure 1.**
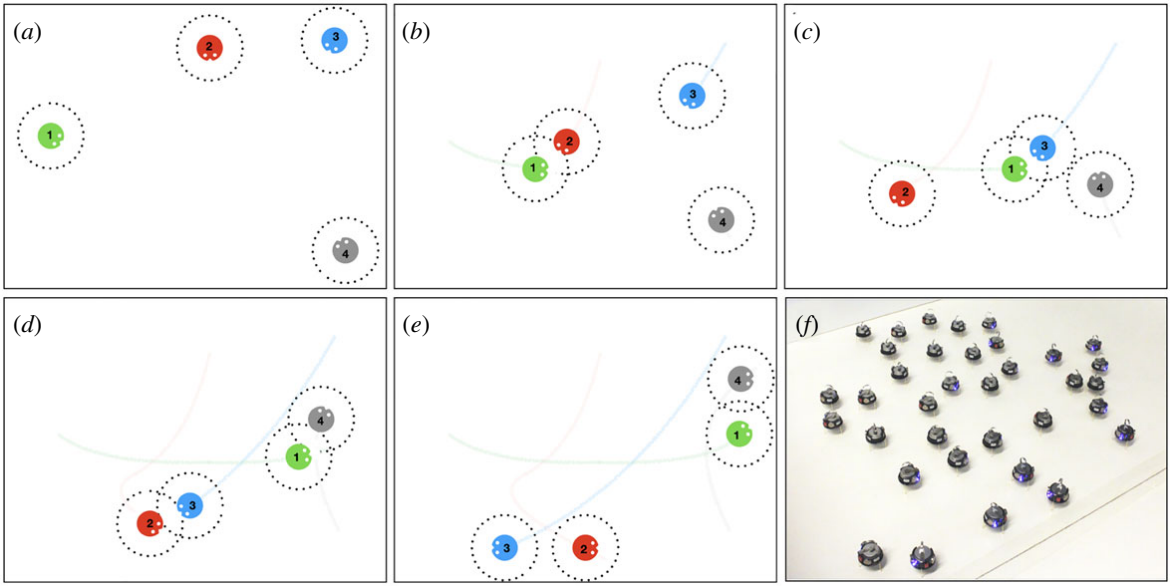
(*a*–*e*) A sequence of snapshots following four robots in a bounded arena. Robots (coloured dots) move in the arena and can exchange information when close enough (communication range for each robot is shown by a dotted circle). Robots are numbered from 1 to 4, each with a particular colour (#1 is green, #2 is red, #3 is blue and #4 is grey). (*a*) Robots start from different locations, then move around (*b*–*e*), possibly crossing the path of other robots (red and green robots in (*b*), green and blue in (*c*), green and grey, as well as red and blue in (*d*)). At the time of the final snapshot (*e*), the green robot experienced more opportunities for communication with other robots, whether or not this robot was the best for the task at hand (which is not specified here). (*f*) A typical swarm of robots used at Sorbonne Université, based on the popular Harvard’s Kilobot robotic platform [26]. Each robot is about 3.3 cm in diameter and can communicate with neighbours at a distance of up to twice its diameter. (Online version in colour.)

Figure 1 illustrates a mock up scenario where four robots navigate the environment and use local sensing and communication. Robots navigate across the arena, possibly fulfilling a task assigned by the human supervisor and interacting with one other robot when they are close enough. In this example, we leave aside the specification of a particular task to focus on the interactions between robots. At each interaction event, robots exchange chunks of information with respect to their behavioural strategy and their current status, which can be anything from their self-assessed performance with respect to the task at hand, to the number of encounters in the past minute. Robots could observe and imitate one another, but can also simply send and receive control parameter values used by their decision-making module. A robot’s task performance may improve over time because its control parameters have been updated with (hopefully better) values from previous encounters.

The green robot, which started from the left of the arena, can be seen as experiencing more opportunities for social learning than any of the three other robots. It also means that this robot’s behavioural strategy had more opportunity to spread to other robots, which is even more likely to occur if this particular behavioural strategy results in high task performance. Depending on the measured efficiency of the three other robots, the green robot’s strategy may spread to all robots if it is estimated to be comparatively more efficient on the task. On the other hand, it will still retain a selective advantage if its efficiency is comparatively average, just because of the larger number of interactions (i.e. comparatively more opportunities for diffusion). In the general case, this entails two kind of selection pressures for the diffusion of a particular behavioural strategy:

— an *implicit selection pressure* that is exogenous and results from interactions of the robots with the environment and with one another. This defines the natural *fitness* of a robot, as understood in biology, in terms of the ability of a robot (aka, the vehicle) to diffuse its own behavioural strategy to other robots, which in the present example is achieved by copying control parameters (see [27,28] for a study of social learning in swarm robotics and natural selection).
— an *explicit selection pressure* that results from the robot’s self-assessment. In this case, self-assessment is performed by using an objective function initially provided by the human supervisor, and which uses information available to the robot to compute its estimated performance with respect to the user-defined task. This results in a quantitative score (e.g. number of harvested items) that can be used to artificially favour or limit the diffusion of the behavioural strategy within the swarm.

As the number of robots in a swarm is finite and (generally) fixed, the way that strategies compete with one another to invade the swarm is similar to the way that Dawkins’ *memes* compete to spread in a population of individuals [29], with successful memes hopping from one robot to another. In the particular case of swarm robotics, diffusion of memes is driven both by the ability to maintain a social network *and* by the ability to fulfill a task that may have *nothing* to do with survivability and/or ability to interact with other robots (e.g. exploration benefits from robots going their separate ways, which hinder interaction between robots).

While the basic principles are presented here, there are of course different ways to implement social learning in swarm robotics. We refer the interested reader to [19] for a comprehensive review.

## 3. Case study: social learning for foraging

In this section, we present a practical example of social learning in swarm robotics. We describe a simulation performed in a pseudo-realistic environment with simple physics [30].^2^ Robots are equipped with distance sensors for local sensing and communication. This roughly corresponds to the use of infra-red devices, which are used to return the distance to nearby obstacles and provide a basic communication apparatus. Each robot uses a control function that maps sensory inputs to motor outputs. Sensory inputs provide information about the distance and type of obstacles nearby (wall, object or robot). Motor outputs are used to set the speed of the left and right motor of a two‐wheel robot. Parameters of the control function are learned using an artificial social learning algorithm. This function computes the left and right motor outputs from sensory inputs: the distance and type of obstacles nearby (wall, object or robot).

In this particular example, we use the horizontal information transfer (HIT) algorithm (see [31] for a full description). With this algorithm, each robot sends a subset of its control parameters along with the current estimation of its performance. The material can be accepted or rejected by the recipient by comparing one’s own self-assessed performance with that received from the other robot. If accepted, the new material is incorporated into the robot’s control function, which essentially means that control parameters are updated with new values. In addition, a mutation event is designed to occur (with low probability) while copying parameter values, which can possibly result in behavioural innovations.

We study a typical foraging task in swarm robotics: robots must capture items, which are spread in the environment. Whenever an item is captured, a new item appears at a random location so that the number of items remains constant over time. A robot’s performance self-assessment is defined as the number of captured items during a period of time (e.g. the past few minutes). For this experiment, we do not use mutation so as to put the emphasis on the dynamics of diffusion of behavioural strategies throughout the population (see Annex for a discussion on the effect mutation). A robot’s behavioural strategy results from a decision-making module that provides motor values as output, computed from sensory inputs (closest wall, robot or object within range). We use a Perceptron (a type of artificial neural network) as control function. A Perceptron is equivalent to a nonlinear weighted combination of input values. There are 98 control parameters per robot.^3^ As shown in the previous section, the Perceptron’s weights (i.e. the control parameters) can be exchanged between robots during an encounter, along with the self-assessed performance estimation of each robot. Hence, imitation is performed directly by copying the control parameters rather than by observing of behaviour, and relies on performance self-assessment to do so.

**Figure 2.**
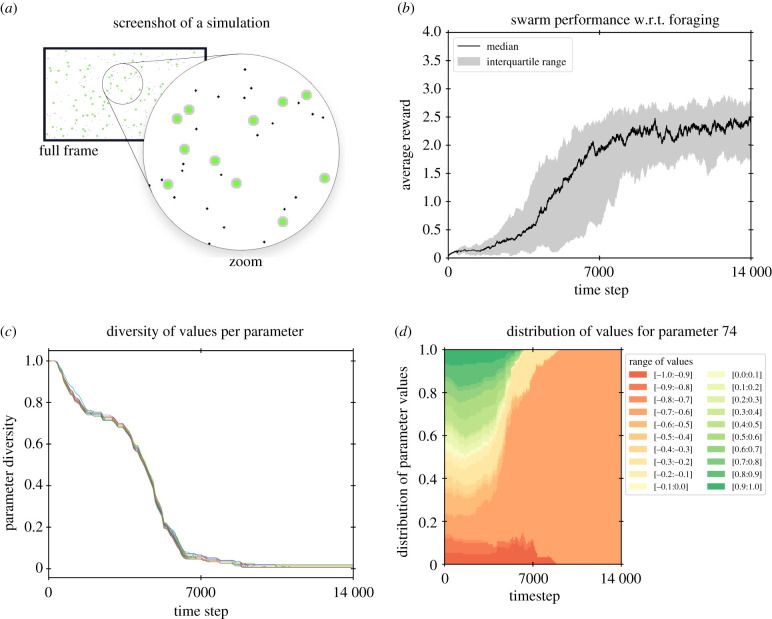
(*a*) The arena (1000×500 units) with 150 robots of diameter 5 (small black circles) and 100 items of diameter 20 (larger green circles). Each robot has 3 × 16 distance sensors distributed uniformly around the body of the robot, each with a range of 16 units (rays not shown). (*b*) Experimental results for 32 independent runs: average reward through time. (*c*) Diversity of parameter values for all control parameters throughout learning in a typical experiment. Diversity is computed as $(1/\left(N_{r}\times N_{p}\right))\sum_{\;p}N_{v|p}$ with a swarm of *N*_*r*_ robots, each robot’s behaviour produced by a decision-making module with *N*_*p*_ control parameters, and with *N*_*v*|*p*_ the number of different unique parameter values *v* for parameter *p* across the *whole* swarm. Maximum diversity (=1.0) is attained when each robot has a unique value for each of its control parameters with respect to (w.r.t.) the whole swarm. Minimal diversity (=1/(*N*_*r*_ × *N*_*v*_)) is attained when all robots in the swarm share the same parameter values for each of the control parameters. (*d*) Example of diffusion of an arbitrarily chosen parameter (parameter no. 74) in a typical experiment. Colours show the distribution of values in the population at different time steps for the selected parameter. The maximum value of 1.0 on the *Y*-axis means all 150 robots use the same value for this control parameter no. 74. w.r.t., with respect to. (Online version in colour.)

Figure 2*a*,*b* show the results compiled from 32 independent experiments. For each experiment, both robots’ and items’ initial positions are randomly picked, as well as random initial values for each robot’s control parameters. The set-up is illustrated in figure 2*a*, with 100 robots and 150 objects that can be collected. The number of objects remains stable over time as a new object appears at a random location whenever a robot captures an existing object. Figure 2*b* shows the average reward per robot over time, compiled from all 100 robots over all 32 independent runs. Reward for one robot is computed as the number of objects captured by a robot in the past 400 time steps. The average performance of individual robots is initially close to zero as initial behavioural strategies perform random movement, and gradually increases to reach a plateau.

All 32 independent runs follow the same dynamics, with qualitatively comparable behavioural strategies, though actual control parameter values may vary at the end of the simulation due to equivalent symmetries in the nature of the neural networks-based controller used. Figure 2*c*,*d* focus on the social learning dynamics for one typical run. Figure 2*c* tracks the diversity of behavioural strategies, using control parameter values as a proxy. Due to random initialization, diversity is close to or equal to its maximum value before learning starts, and decreases through time due to diffusion of efficient behavioural strategies, ending with a unique parameter value for each of the control parameters among the whole population of a given run. In this particular run, it can be seen that diffusion of parameter values is not monotonically decreasing, as a first plateau is reached and maintained for some time. Diversity then decreases again until a final plateau is reached, close to the minimal value for diversity (and maximal value for performance). Figure 2*d* completes this analysis by focusing on a particular (randomly chosen) control parameter for this same experiment: the current values for this parameter among all robots of the swarm are grouped by interval and tracked through time. Here, values in the range [−0.7, −0.6] compete with others and finally invade the whole population, ultimately ending up with one unique value in this range (not visible here).

**Figure 3.**
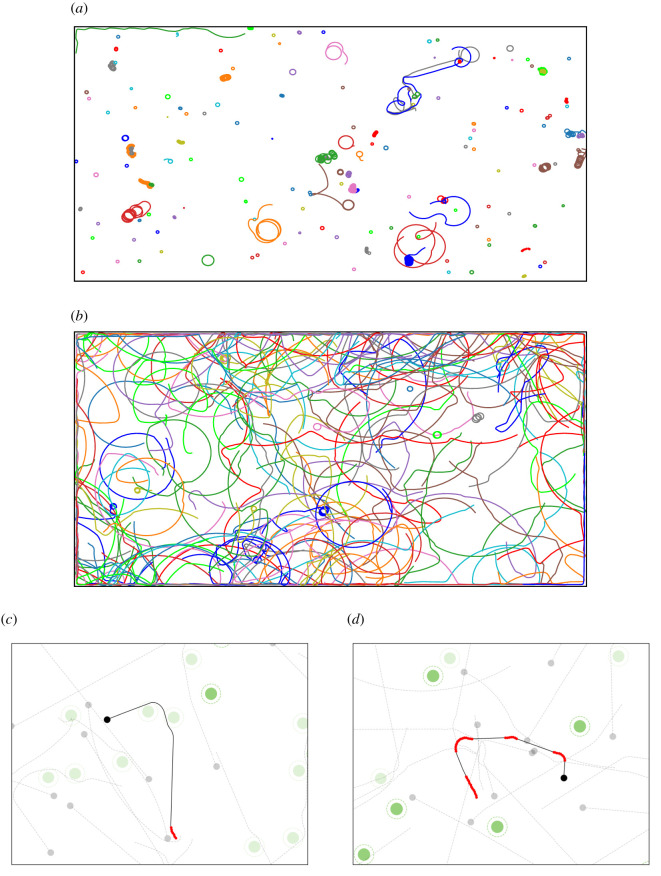
(*a*) Initial trajectories of 150 robots in the first time steps of learning (0−400 time steps, items are not displayed). (*b*) Trajectories of 150 robots at the end of the simulation (13 600–14 000 time steps). (*c*,*d*) Two examples of robot trajectories produced after learning, for an arbitrary selected focal robot (small black circle) and its trajectory during the last 100 time steps (black or red line—red denotes time steps where the focal robot shared information with a nearby robot). The figures also show other robots (small grey dots) and items (large green dots—lighter green denotes an item that has been captured by a robot during the 100 time steps).

Actual behaviours for this typical run are shown in figure 3. At the very beginning, robots fail to either meet with one another or to capture objects other than by chance (figure 3*a*). At the end of learning, robots wander around, covering large areas and following non-linear trajectories (figure 3*b*). Trajectories from typical behavioural strategies are illustrated in figures 3*c*,*d*. Each figure part displays the trajectory of one robot and shows its interaction with other robots and objects (e.g. the robot turns toward a detected object). These two figure parts illustrate the result of both the implicit exogenous selection pressure (robots meet with one another as it favours diffusion of behavioural strategies) and the explicit endogenous selection pressure (robots are drawn to items as this increases their total reward).

While this experiment illustrates social learning in a robot swarm in ideal conditions,^4^ it is important to note that the behavioural outcome depends on the task and the environment at hand. First, behavioural strategies would be different for another task (e.g. collective object transport, or search and rescue). Second, swarm density may completely change the swarm dynamics, even if the environment and task remained the same (see [32] for a discussion on the impact of social network topology on collective cognition).

## 4. Discussion: future of social learning in swarm robotics

As discussed in the previous sections, the goal with social learning in swarm robotics is to optimize the efficiency of the swarm *as a whole*, as performance is ultimately measured with respect to a user-defined task (see [19] for a comprehensive review of recent works). Swarm robotics is distributed by definition, which raises the question of providing an accurate estimation of the contribution of each individual to the performance of the collective. Such contribution is evaluated individually, by each robot, based on its immediate perception of the surroundings, which implies that evaluation may be partial. By carefully defining a performance function before deployment, it may be possible to accurately evaluate the benefit of one robot’s action, but unless the environment is perfectly known (which it is not) this is generally unattainable. To use our previous example of a foraging task, having all robots looking for items makes sense, but could be detrimental in a cluttered environment where robots would spend their time avoiding collisions with each other and competing for resources. This corresponds to the problem of estimating the individual’s *marginal contribution* in a group, which is well known in the field of collective intelligence in AI [33]. Compared to natural systems, swarm robotics decision making is designed rather than learned: it is thus possible to define rules so that individuals behave the way we would want them to [34]. For example, an individual’s score could depend on how well its neighbours are faring, thus indirectly favouring altruistic behaviours.

Now, let us consider social learning from a broader perspective, where the distinction between artificial and natural collective systems is less evident. Current swarm robotics social learning algorithms implement cumulative culture evolution, by repeating the process of introduction, transmission and improvement of new behavioural strategies. However, this is a rather crude implementation compared to what is observed in nature [35–37], as all robots in the swarm are both physically and logically identical: all robots are interchangeable and would take a similar decision if presented with the same sensory stimulus. Implementing or learning a complex social structure (e.g. leader election or partner choice [38]) as well as introducing physically distinct types of robot hardware may benefit a robot swarm’s ability to handle challenging environments and problems, by enabling cooperation and division of labour (see [39] in this issue for a discussion on the benefits of a multi-level social structure in social learning). Also, individual robots are currently limited to displaying reactive behaviours (feed-forward neural networks or decision trees, see [19]) and basic social interactions based on direct information transfer (sharing control parameters in one form or another). Transitioning towards more cognitive decision making (e.g. learning and combining model-free and model-based decision making [40]) could enable more complex coordinated behaviours (see also [41] for similar concerns within a small group of robots). For example, capabilities such as shared-intentionality or reason-giving similar to what humans do (see [42] in this issue) could enable more subtle ways of interacting and performing social learning.

In this section, we focused on challenges that are specific to social learning that, if solved, would improve the *outcome* of social learning.^5^ For the sake of completeness, we should also mention that major challenges are yet to be addressed in swarm robotics in general, i.e. not limited to learning. The interested reader is referred to [4,47,48] for recent discussions on hardware, software or operational issues raised in swarm robotics.

## 5. Conclusion

This paper discussed the deployment of social learning algorithms in robot swarms. This is a rather particular instance of machine learning methods that is applied to distributed robotics systems where sensing, decision making and performance evaluation are performed locally. As presented throughout this paper, the formulation of social learning in swarm robotics aims at achieving a task *after* robots are actually deployed. However, this artificial instantiation of social learning also shares a lot with its natural counterpart. In particular, cumulative culture evolution enables the emergence of complex social behaviours (e.g. division of labour or cooperation) by gradually accumulating useful behavioural skills.

The main motivation behind investigating social learning in swarm robotics stems from the fact that many real-world situations *cannot* be modelled beforehand. This can be the case for several applications where swarm robotics is expected to play a role [4], such agriculture robotics (e.g. small robots for monitoring, pest destruction or pollinating) or biomedical applications (e.g. drug delivery and bio-sensing), for the mere reason that real-world physics is *extremely* challenging to model, even in a simple controlled setting [10]. As recently noted by several authors [49–52], dense robot swarms, where many physical collisions occur, can be considered from a statistical physics perspective as an active matter. The benefits of physical interactions may then be identified and exploited (e.g. particle alignment [53]) while learning to solve the task.

A final remark should be made about the use of robotics systems beyond engineering. If we leave aside the engineering *raison d’être* of swarm robotics, we are left with an artificial system that implements social learning under realistic constraints. Robots, just like animals, sense, act and communicate with their neighbours, and each robot may discover new behaviours or copy behaviours from others. Similar to the use of computational and robotics simulations for studying natural evolution [54], the study of social learning in nature may benefit from a modelling and simulation tool more closely matching real world conditions [55,56]. It would nicely complement observation, mathematical modelling and computational simulation of social learning processes.

## Annex

**Figure 4.**
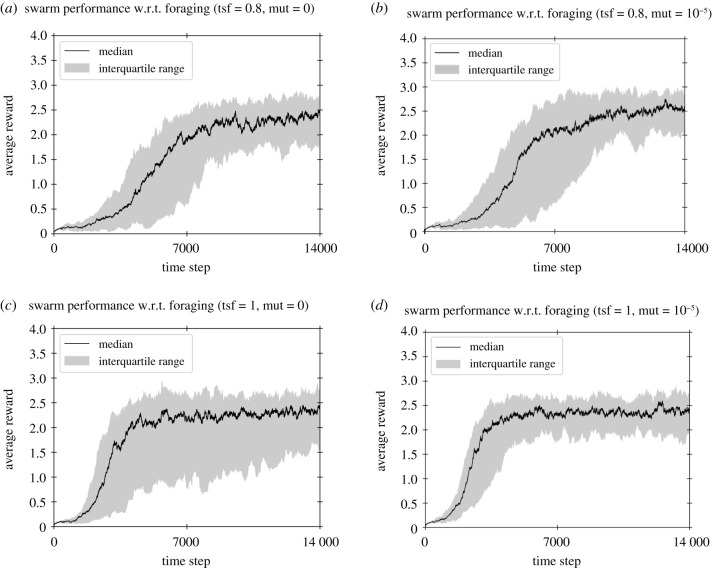
Comparison between setups with different transfer and mutation settings. Transfer accounts for the proportion of control parameter values that are exchanged between robots. tsf = 0.8 and tsf = 1.0 respectively signify that 80% and 100% of the parameter values are sent during an encounter between robots. Mutation accounts for the probability that an individual’s control parameters are re-initialized. Mutation is either deactivated (mut = 0), or set to 10^−5^, which corresponds to resetting one robot among 150 every 400 time steps (on average). (*a*–*d*) Results for four different settings, each with their own transfer volume and mutation rate, using 32 independent runs per setup. Panel (*a*) is identical to what was presented earlier in figure 2. mut, mutation rate; tsf, transfer volume; w.r.t., with respect to.

This Annex complements results presented in §3. We quickly discuss the effect of mutation, which was inactivated previously in order to emphasize the diffusion of information over innovation for which *de novo* mutation plays an important role. Figure 4 shows a comparison between different setups, each with a particular transfer volume and mutation rate. While transfer refers to the amount of information that is sent from one robot to another during an encounter, mutation refers to the amount of perturbation that may spontaneously occur in the population.

Figure 4*a*–*d* provides two insights. First, a transfer volume of *α* = 0.8 without mutation (figure 4*a*) shows a reduced variance between runs compared to *α* = 1.0 without mutation (figure 4*c*), even though transferring larger volume with *α* = 1.0 shows initial faster learning. Similarly, a mutation rate of *σ* = 10^−5^ also reduces the variance between runs using a similar transfer volume (figures 4*a* versus *b*, and 4*c* versus *d*). Indeed, both transfer and mutation may generate novel behavioural strategies, which can be beneficial in terms of efficiency, or at least mitigate a pool of poor random initial strategies. Transfer with *α* < 1.0 enables recombination of part of the existing control parameter sets, though it slows down convergence speed (figure 4*a*,*b* versus 4*c*,*d*). Similarly, mutation with probability *σ* > 0 allows for the introduction of new control parameter values, which makes it possible to diversify the reservoir of parameter values initially present in the swarm, and possibly lead to new and more efficient behavioural strategies.

The interested reader is referred to [31] for a comprehensive analysis of the effect of transfer and mutation on convergence speed and performance.

## Endnotes

1 The general algorithmic idea of social learning in swarm robotics was introduced in [21,22] where the term ‘embodied evolution’ is used to refer to the nature of the method. The term ‘embodied’ is to be understood here as referring to the implementation of the evolutionary algorithm within the robots, rather than evolutionary learning being conducted by an external computer that orchestrates learning and monitors performance for every robot in a centralized manner. The first actual use of the term ‘social learning’ in swarm robotics is found in [23,24], with earlier use in evolutionary robotics appearing in [25]. Its use is preferable as it emphasizes the nature of the learning performed, and leaves open the method used to actually perform learning.

2 The use of simulation in this paper makes it possible to perform a large scale experiment while monitoring both the full trajectories and internal states, which is difficult in a real setup. Experimental parameters have been chosen to provide a typical example of social learning. The interested reader is referred to [28] for experiments with real robots and extensive parameter sensitivity analysis, including the transfer to real robots of an algorithm first evaluated in the simulator used here.

3 Technical details: we use an artificial neural network without a hidden layer. Output neurons use a hyperbolic tangent activation function. Inputs: three groups of 16 sensors, distributed uniformly around the robot. Each of the three groups of sensors returns the distance to either the closest object, wall or robot. Sensors are of limited length (approx. 3 times the robot’s body length) and normalized between 0.0 and 1.0. Each sensor returns a maximal value if nothing is detected. An additional input is added of value 1.0 (the bias node). Outputs: translation and rotation speed. Weight initialization is performed randomly in [ − 10, + 10].

4 i.e. The environment is neither too dense nor too sparse in terms of number of robots and resources.

5 Other important challenges such as AI explainability [43–45] and ethical issues [46] are voluntarily left aside as they do not offer *per se* a means to improve social learning w.r.t. pure performance.

## Data accessibility

Source code for all experiments in this paper is accessible at https://github.com/nicolasFontbonne/HIT_pyroborobo.
